# Clinical characteristics and role of whole-body bone scan in multifocal osteonecrosis

**DOI:** 10.1186/s12891-019-2401-y

**Published:** 2019-01-15

**Authors:** Young-Sil An, Sunghoon Park, Ju-Yang Jung, Chang-Hee Suh, Hyoun-Ah Kim

**Affiliations:** 1 Department of Nuclear Medicine and Molecular Imaging, Ajou University School of Medicine, Suwon, Korea; 2 Department of Radiology, Ajou University School of Medicine, Suwon, Korea; 3 Department of Rheumatology, Ajou University School of Medicine, Suwon, Korea

**Keywords:** Osteonecrosis, Whole-body bone scan, Magnetic resonance imaging, Risk factor

## Abstract

**Background:**

Multifocal osteonecrosis (ON) is defined as ON involving three or more distinct anatomical sites. We investigated the clinical characteristics and utility of whole-body bone scans (WBBS) in patients with multifocal ON.

**Methods:**

A total of 254 patients with ON confirmed by magnetic resonance imaging (MRI) or X-rays of the hips or other anatomic regions were evaluated using WBBS and divided into those with multifocal disease and those with oligofocal disease; their clinical characteristics were then compared. All data were analyzed retrospectively both visually and quantitatively (via uptake grading and defect scoring). Associations between the MRI Association Research Circulation Osseous (ARCO) classification and bone scan photon defects and uptake grade were assessed. Factors associated with multifocal ON were identified using logistic regression.

**Results:**

Of the 254 ON patients, 26 (10.2%) had multifocal ON. Their mean age (42.8 ± 14.3 years) was less than that of patients with oligofocal ON (50.9 ± 15.4 years; *p* = 0.011). Comorbidities, corticosteroid use, and treatment with immunosuppressive agents were more frequent in patients with multifocal ON. Age (odds ratio [OR] = 0.964, *p* = 0.013), the presence of a comorbidity (OR = 3.387, *p* = 0.006), present corticosteroid use (OR = 5.696, *p* < 0.001), and treatment with immunosuppressive agents (OR = 3.447, *p* = 0.004) were significantly associated with multifocal ON. The MRI ARCO classification was not associated with photon defects in the bone scans of those with femoral ON. However, the ARCO classification was significantly associated with uptake grade.

**Conclusions:**

WBBS may be an additional tool for evaluating ON patients with risk factors for multiple ON, such as younger age, corticosteroid use, and comorbidities.

## Background

Osteonecrosis (ON) is a serious disease that causes joint pain and significant physical disability. Various joints can be affected, including the shoulders, knees, and ankles, but the most commonly affected site is the hip [1, 2]. Although the detailed pathogenesis of ON remains unclear, ON may reflect bony ischemia caused by direct injury or vascular damage [3]. Trauma, excessive alcohol intake, corticosteroid treatment, and rheumatic and malignant diseases contribute to the development of ON [4]. Multifocal ON, which ON involves three or more distinct anatomical sites [5], is rare, being seen in only approximately 3% of all ON patients [5]. Corticosteroid use is a known risk factor for multifocal ON [5, 6], as are certain comorbidities, including systemic lupus erythematosus (SLE), renal failure, leukemia, and lymphoma [5, 7, 8]. However, almost all studies of multifocal ON are case reports and case series, so the incidence and clinical characteristics of the condition remain poorly defined [5, 8–13].

Imaging is used to diagnose ON and evaluate the severity of lesions. Radiographs are often used initially, but early-stage disease may not be detected because radiographic abnormalities develop only after prolonged ischemic change [14]. Magnetic resonance imaging (MRI) detects ON with high sensitivity and is used to assess the severity of the disease [15]. However, routine MRI does not cover all joints because of the high cost. A whole-body bone scan (WBBS) is more sensitive than a simple X-ray in terms of diagnosing ON [16–18]. A WBBS can be used to screen for multifocal ON, revealing abnormal bone uptake of Tc-99 phosphates [19]. However, bone scintigraphy is of low sensitivity when used to diagnose symptomatic ON [1]. Although several studies have reported bone scan data for ON patients, the results are inconsistent and limited to case reports or case series with small numbers of patients [1, 16–19].

Therefore, we retrospectively reviewed the medical records of ON patients and their WBBS, X-ray, and MRI data; identified those with multifocal ON; and investigated the clinical characteristics and utility of WBBS in patients with multifocal ON.

## Methods

### Subjects

We identified 294 patients among the computerized medical records held at Ajou University Hospital with the diagnostic code for ON and test code for WBBS; 40 patients were excluded because their diagnosis was not confirmed to ON and MRI findings were negative. We retrospectively reviewed data on 254 patients with ON confirmed by MRI or X-ray of the site at the time of their initial visit between 2003 and 2017. We reviewed the WBBS and the MRI or X-rays of the sites, such as hips, knees, ankles and shoulders. We divided the patients into multifocal and oligofocal ON groups. Multifocal ON was defined as ON involving three or more separate anatomical sites, as described previously [5]. A total of 26 patients diagnosed with multifocal ON were compared to the 228 remaining patients with a diagnosis of oligofocal ON. The study was approved by our institutional review board (approval no. AJIRB-MED-MDB-18-041).

### Variables

All clinical data were retrieved from medical records stored in the hospital database. Age, sex, all comorbidities, clinical symptoms and their duration, and previous treatment were recorded at the time of WBBS. The cumulative corticosteroid dose (prednisolone equivalent for all oral, intravenous, subcutaneous, and intramuscular administrations) during the WBBS was calculated. Excessive alcohol intake was defined as consumption of more than 400 mL of alcohol per week. Soju is a distilled Korean liquor; one bottle of Soju contains 72 mL of alcohol (i.e., 360 mL containing 20% alcohol). Therefore, excessive alcohol intake can be defined as consumption of one bottle of Soju per day, or two bottles of Soju on 3 or more days per week. We divided the causes of ON into four categories: 1) idiopathic, 2) trauma, 3) a comorbidity requiring the use of corticosteroids or immunosuppressive agents, and 4) excessive alcohol intake.

### WBBS acquisition

WBBS was performed 4 h after injection of 740 MBq technetium-99 m hydroxymethylene diphosphonate (Tc-99 m HDP). Anterior and posterior views were acquired using a double-headed gamma camera equipped with a VariCam Millennium VG low-energy high-resolution collimator (GE Medical Systems, Milwaukee, WI, USA). Images were analyzed on a Xeleris Workstation (GE Healthcare, Buckinghamshire, UK).

### WBBS image interpretation and analysis

All WBBSs were analyzed visually by a specialist in nuclear medicine, with 14 years of experience (Y.S.A.), blind to all other clinical data. Bone regions exhibiting uptake were recorded, and the extent of the uptake was graded visually from 0 to 2 (0 = no uptake, 1 = mild, 2 = intense). In addition, uptake was recorded as bilateral or unilateral. Photon defects were also noted.

### Simple radiographs and MRI

Simple radiographs and MR images were obtained using standard imaging protocols featuring at least two projections or planes. MR imaging was performed using a 1.5-T (Signa HDxt, Signa Excite; GE Healthcare) or a 3 T (Achieva; Philips Healthcare, Best, The Netherlands) system fitted with commercial body or extremity coils depending on the location of the lesion. All sequences included fat-saturated T2-weighted and T1-weighted images (non-contrast T1 and fat-suppressed contrast-enhanced T1 images).

### Simple radiograph and MR image interpretation and analysis

A musculoskeletal radiologist with 10 years of experience (S.P.), blind to the clinical information evaluated simple radiographs and MR images. On a simple radiograph, ON was defined as a combination of sclerosis, cystic change, and a crescent-shaped, subchondral, lucent lesion [20]. On an MR image, a band-like, crescent-shaped, or sector-like region of bone marrow replacement and the double-line sign (an inner line of high signal intensity running parallel to an outer line of low signal intensity) were considered diagnostic of ON, which was classified using the Association Research Circulation Osseous (ARCO) system [21].

### Statistical analyses

All statistical analyses were performed using SPSS software (ver. 23.0; SPSS Inc., Chicago, IL, USA). *p* < 0.05 was considered to reflect statistical significance. Results are presented as means ± standard deviation (SD) for continuous variables and as frequencies with percentages for categorical variables. Independent Student’s t-test was used to compare continuous variables, and Pearson’s chi square test was used to compare categorical variables. Factors associated with multifocal ON were defined using logistic regression. The extent of the agreement between WBBS and hip MRI or X-ray images was evaluated using the kappa (κ) statistic; κ >  0.8 represents excellent agreement, 0.61–0.8 good agreement, 0.41–0.6 moderate agreement, 0.21–0.4 fair agreement, and < 0.2 poor agreement [22].

## Results

### Clinical characteristics of patients with multifocal and oligofocal ON

**Fig. 1:**
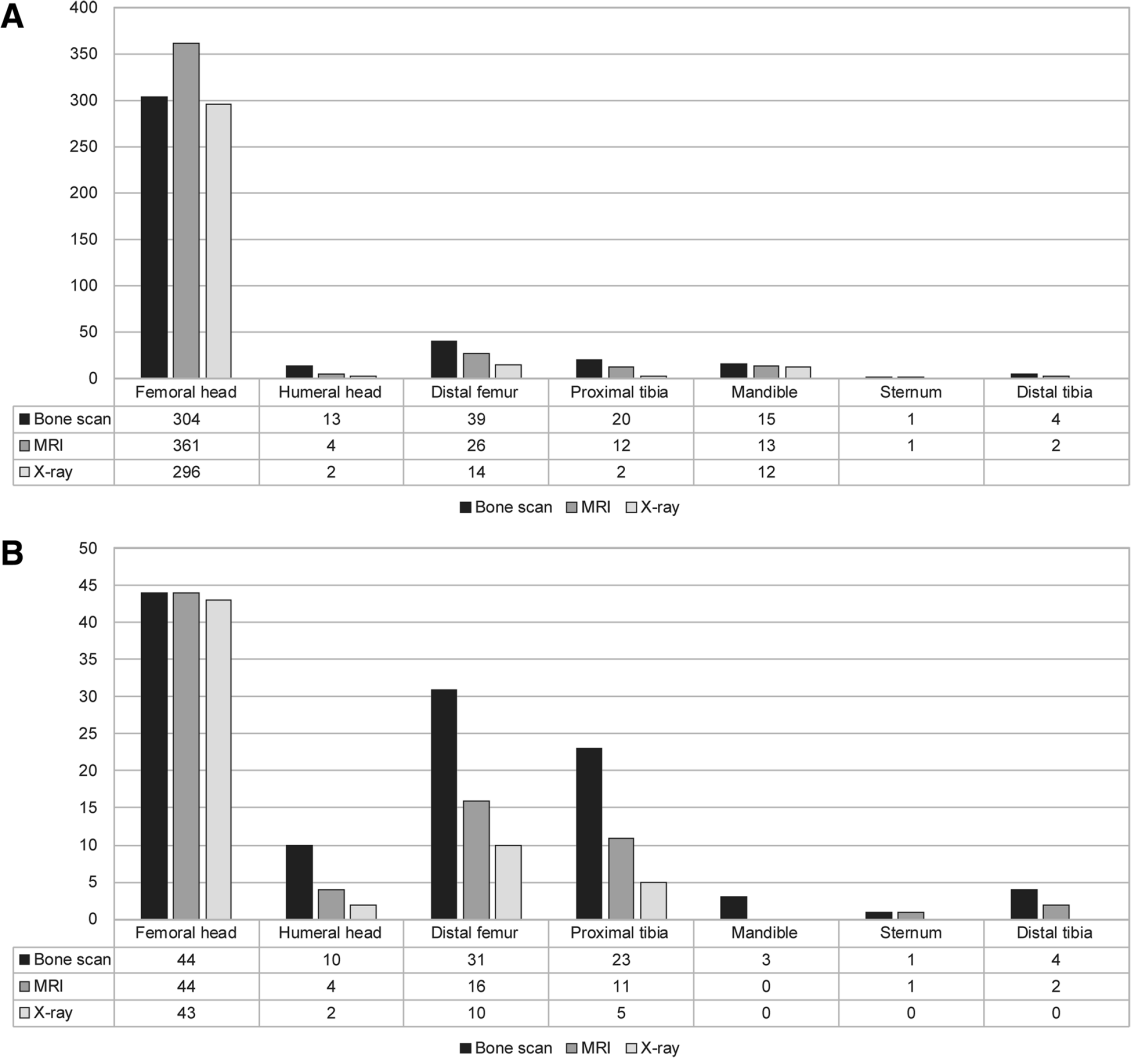
**a** Osteonecrosis (ON) sites confirmed by whole body bone scan (WBBS) and magnetic resonance imaging (MRI) or X-ray in 254 patients with ON. **b** ON sites confirmed by WBBS and MRI or X-ray in 26 patients with multiple ON. Local MRI was obtained for 419 sites and X-ray images of 415 sites were obtained in the 254 patients with ON. Overall, WBBS images of 477 sites were compared with local MR or X-ray images

Table 1 lists the clinical characteristics of the 254 patients with ON confirmed by MRI or X-ray. Their mean age was 50.0 ± 15.4 years, and 152 (59.8%) were male. A total of 26 patients (10.2%) had multifocal ON as shown by WBBS and MRI or X-ray. Twenty-four patients had multifocal ON evident on WBBS and MRI or X-ray. In the other two patients, multifocal ON was confirmed by multifocal site evaluation by MRI or X-rays, with negative results on WBBS. The mean age of patients with multifocal ON (42.8 ± 14.3 years) was less than that of patients with oligofocal ON (50.9 ± 15.4 years; *p* = 0.011). Accompanying comorbidities were more common in patients with multifocal ON (*n* = 18, 69.2%) than oligofocal ON (*n* = 91, 39.9%; *p* = 0.006). Current corticosteroid use was more common in those with multifocal ON (16, 61.5%) than oligofocal ON (50, 21.9%; *p* < 0.001). However, cumulative corticosteroid dose did not differ between the groups (*p* = 0.859). Immunosuppressive agents were more commonly taken by patients with multifocal ON (11, 42.3%) than oligofocal ON (40, 17.5%; *p* = 0.007). Table 2 lists the causes of ON. The most common cause of multifocal ON was a comorbidity requiring the use of corticosteroids or immunosuppressive agents (18, 69.2%); the other causes were idiopathic (4, 15.4%), alcohol intake (3, 11.5%), and trauma (1, 3.8%). The most common cause of oligofocal ON was excessive alcoholic intake (74, 32.5%), followed by a comorbidity requiring the use of corticosteroids or immunosuppressive agents (70, 30.7%), idiopathic causes (60, 26.3%), and trauma (24, 10.5%). The most common cause of ON thus differed for multifocal and oligofocal ON (*p* = 0.001). Table 3 lists the comorbidities in all patients. Figure 1 shows ON sites confirmed by WBBS and MRI or X-rays in all 254 patients.

**Table 1:** Clinical characteristics of multifocal and oligofocal osteonecrosis (ON) in 254 patients with ON

	Multifocal ON (*n* = 26)	Oligofocal ON (*n* = 228)	Any ON (*N* = 254)	*p*-value
Age (years)	42.8 ± 14.3	50.9 ± 15.4	50.0 ± 15.4	0.011
Sex (M/F)	15 (57.7)/11 (42.3)	137 (60.1)/91 (39.9)	152 (59.8)/102 (40.2)	0.835
Smoking (yes)	7 (26.9)	94 (41.2)	101 (39.8)	0.205
Alcohol use (excessive)	10 (38.5)	104 (45.6)	114 (44.9)	0.538
Diabetes mellitus	3 (11.5)	11 (4.8)	14 (5.5)	0.162
Hypertension	10 (38.5)	54 (23.7)	64 (25.2)	0.150
Trauma	1 (3.8)	37 (16.2)	38 (15.0)	0.007
Symptom sites (n)	1.92 ± 1.26	1.26 ± 0.44	1.33 ± 0.61	0.014
Symptom duration (months)	7.0 ± 9.3	7.5 ± 13.7	7.4 ± 13.3	0.855
Comorbidity	18 (69.2)	91 (39.9)	109 (42.9)	0.006
Comorbidity duration (years)	6.37 ± 6.77	6.74 ± 6.80	6.68 ± 6.77	0.833
Use of CS	16 (61.5)	50 (21.9)	66 (26.0)	< 0.001
Maximal dose of CS	284.4 ± 427.3	154.0 ± 317.0	185.6 ± 347.9	0.273
Cumulative dose of CS	5310.7 ± 5359.6	5704.8 ± 7664.7	5612.8 ± 7153.0	0.859
Use of immunosuppressive agents	11 (42.3)	40 (17.5)	51 (20.1)	0.007
Osteoporosis	1 (3.8)	12 (5.3)	13 (5.1)	> 0.999
Use of BP	1 (3.8)	10 (4.4)	11 (4.3)	> 0.999
BP duration (months)	24	49.7 ± 18.5	47.4 ± 19.2	
ON sites confirmed by X-ray and MRI	3.04 ± 1.42	1.52 ± 0.53	1.68 ± 0.81	< 0.001
ON sites confirmed by bone scan	4.15 ± 2.05	1.27 ± 0.53	1.57 ± 1.20	< 0.001

*ON* osteonecrosis, *CS* corticosteroid, *BP* bisphosphonate, *MRI* magnetic resonance imaging. All values are presented as numbers (%) or means ± SD. Independent Student’s *t*-test was used to compare continuous variables, and Pearson’s chi square test was used to compare categorical variables between multifocal and oligofocal ON

**Table 2:** Causes of multifocal and oligofocal osteonecrosis (ON) in patients with ON

Cause of ON	Multifocal ON (*n* = 26)	Oligofocal ON (*n* = 228)	Any ON (*N* = 254)	*p*-value
Idiopathic	4 (15.4)	60 (26.3)	64 (25.2)	0.001
Trauma	1 (3.8)	24 (10.5)	25 (9.8)	
Comorbidity requiring corticosteroids and immunosuppressive agents	18 (69.2)	70 (30.7)	88 (34.6)	
Excessive alcohol intake	3 (11.5)	74 (32.5)	77 (30.3)	

*ON* osteonecrosis. Pearson’s chi square test was used to compare causes of multifocal and oligofocal ON

**Table 3:** Comorbidities in patients with multifocal and oligofocal osteonecrosis (ON)

Comorbidity	Multifocal ON (*n* = 18/26)	Oligofocal ON (*n* = 70/228)	Any ON (*N* = 88/254)
**Immune system disease**	**8**	**26**	**34**
Ulcerative colitis	1		1
Rheumatoid arthritis		5	5
AOSD		1	1
Systemic lupus erythematosus	3	7	10
MCD, MGN, IgA nephropathy	2	7	9
ANCA-associated vasculitis	1	1	2
Dermatomyositis		3	3
Sarcoidosis, Behçet’s disease, vitalism	1	2	3
**ESRD with KT**	**3**	**6**	**9**
**Malignancy**	**2**	**16**	**18**
Lung cancer		1	1
Stomach cancer	1	2	3
Renal cancer		2	2
Breast cancer	1	5	6
Neuroblastoma		2	2
Rectal cancer		2	2
Gall bladder cancer		1	1
Hepatocellular carcinoma		1	1
**Hematological diseases**		**4**	**4**
Acute myelocytic leukemia		1	1
Multiple myeloma		1	1
Aplastic anemia		2	2
**Mitral valve stenosis, with operation**		**1**	**1**
**Spinal cord tumor, meningioma, pheochromocytoma**	**1**	**2**	**3**
**Craniopharyngioma, seizure**	**1**	**1**	**2**
**Osteoporosis with bisphosphonate treatment**		**2**	**2**
**Bronchiectasis**		**1**	**1**
**Facial palsy, hearing loss, trigeminal neuralgia**	**1**	**2**	**3**
**Recurrent oral ulcer, recurrent uveitis**	**1**	**1**	**2**
**Allergic disease (urticarial, Steven-Johnson), vitalism, asthma**	**1**	**5**	**6**
**Liver cirrhosis, intraperitoneal bleeding**		**3**	**3**

*ON* osteonecrosis, *AOSD* adult onset Still’s disease, *MCD* minimal change disease, *MGN* membranous glomerulonephritis, *ANCA* anti-neutrophil cytoplasmic antibody, *ESRD* end-stage renal disease, *KT* kidney transplantation

### Factors associated with multifocal ON

**Fig. 2:**
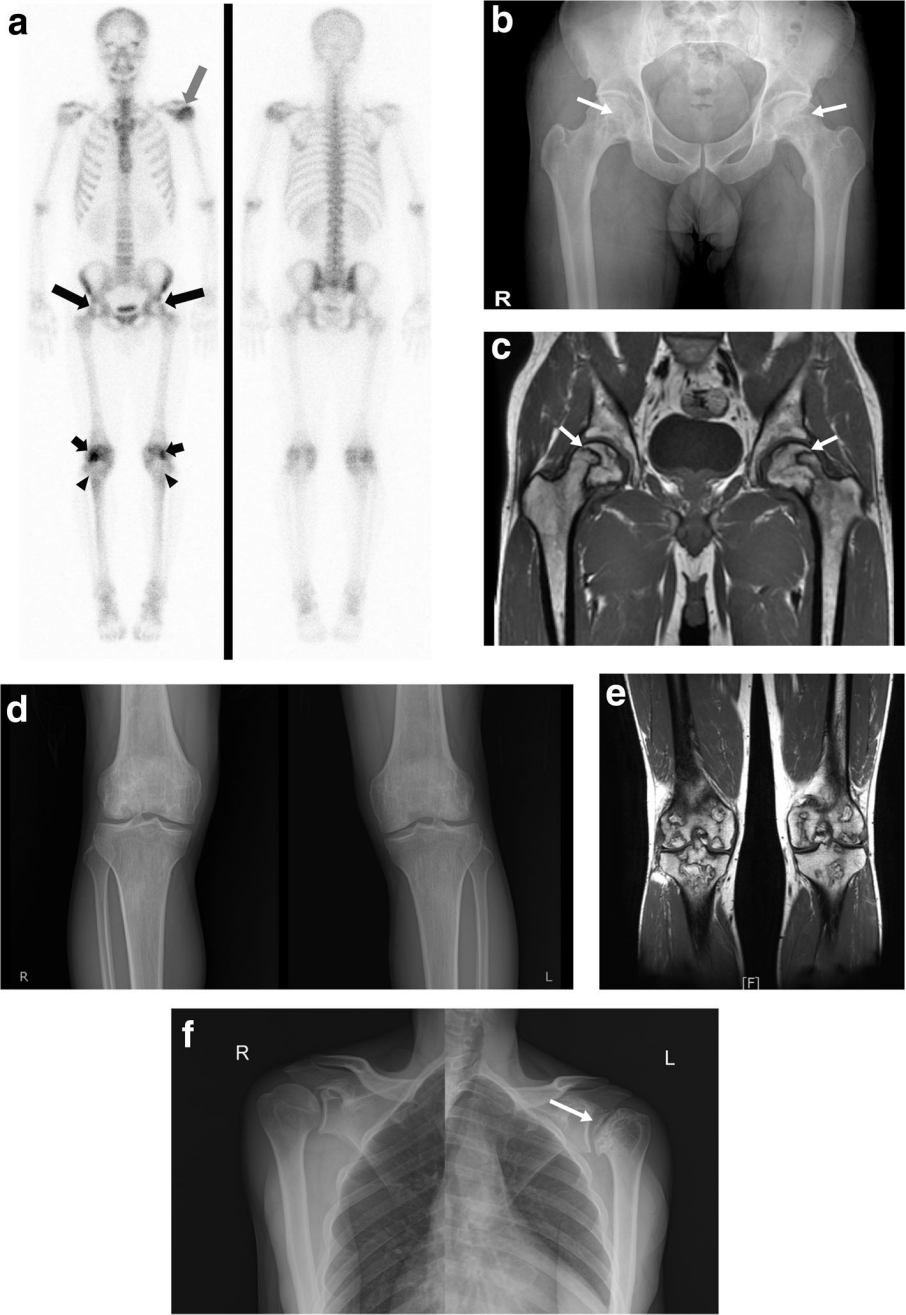
A 42-year-old male case of multifocal osteonecrosis (ON). **a** This is a bone scintigraphy performed in a 42-year-old male patient with abnormal photon-defects in both femoral heads (long arrows). Also abnormal hot uptakes were also observed in the left humeral head (white arrow), both distal femoral (short arrows) and proximal tibial regions (arrowheads), suggesting that AVN is suspected. **b** Pelvis anteroposterior view (AP) shows radiolucency and surrounding sclerosis of both femoral heads (arrows). **c** Coronal T1-weighted image shows large areas of low signal intensity demonstrated typical band-like pattern of ON involving both femoral heads (arrows). **d** Both knee AP views show ill-defined lucency and sclerosis with cyst like appearance of both distal femur and proximal tibia. **e** Coronal T1-weighted image shows multiple geographic lesions with peripheral low signal intensity of rim involving both distal femur and proximal tibia. **f** Simple radiographs show more advanced degree of ON with subchondral fracture and mild osteoarthritic change involving left humeral head (arrow)

We evaluated factors associated with multifocal ON (Table 4). Age (odds ratio [OR] = 0.964, *p* = 0.013), comorbidity (OR = 3.387, *p* = 0.006), corticosteroid use (OR = 5.696, *p* < 0.001), and treatment with immunosuppressive agents (OR = 3.447, *p* = 0.004) were significantly associated with multifocal ON in univariate analyses. Age (OR = 0.967, 95% confidence interval [CI]: 0.940–0.994, *p* = 0.017) and comorbidity (OR = 2.674, 95% CI: 1.033–6.922, *p* = 0.043) were significantly associated with multifocal ON in a multivariate logistic regression that also evaluated alcohol intake and trauma. Corticosteroid use (OR = 4.512, 95% CI: 1.702–11.964, *p* = 0.002) was significantly associated with multifocal ON in a multivariate logistic regression that also included alcohol intake, trauma, and corticosteroid use. However, only age (OR = 0.969, 95% CI: 0.941–0.997, *p* = 0.028) was associated with multifocal ON in multivariate analyses that included age, alcohol intake, trauma, and the use of immunosuppressive agents. Figure 2 shows the WBBS, MRI, and X-ray of a case of multifocal ON.

**Table 4:** Logistic regression analyses of factors associated with multifocal osteonecrosis (ON) in patients with ON

Clinical factor	Univariate			*p*-value	Multivariate			*p*- value	Multivariate			*p*- value
	OR	95% Wald CI			OR	95% Wald CI			OR	95% Wald CI		
Age	0.964	0.937	0.992	0.013	0.967	0.940	0.994	0.017	0.978	0.950	1.008	0.149
Alcohol abuse	0.745	0.324	1.712	0.488	0.894	0.360	2.219	0.810	1.131	0.438	2.917	0.799
Trauma history	0.206	0.027	1.571	0.128	0.273	0.034	2.188	0.273	0.257	0.033	2.024	0.197
Comorbidity	3.387	1.413	8.118	0.006	2.674	1.033	6.922	0.043	–			
Use of corticosteroids	5.696	2.434	13.328	< 0.001	–				4.512	1.702	11.964	0.002
Use of immunosuppressive agents	3.447	1.474	8.061	0.004	–				–			

*ON* osteonecrosis

### WBBS, MRI, and X-ray results

Both WBBS and local MRI images were obtained for 458 sites, and 456 sites were evaluated with WBBS and X-ray images among the 294 patients who underwent WBBS with a code of ON. Therefore, a total of 458 sites were compared based on WBBS and MR images, and 456 were compared based on WBBS and X-ray images (Table 5). WBBS was positive for 346 sites (75.5%), and the MRI for 419 (91.4%). Of the 458 sites, WBBS and MRI were in agreement for 369. The κ coefficient of agreement between WBBS and MRI data was fair (κ = 0.325, *p* < 0.001). WBBS was positive for 348 (76.3%) of the 456 sites, and the X-rays for 326 (71.4%). Of the 456 sites, WBBS and X-rays were in agreement for 362. The coefficient of agreement was moderate (κ = 0.467, *p* < 0.001).

**Table 5:** Agreement between whole-body bone scan (WBBS) data and magnetic resonance imaging (MRI) and X-ray data in patients with osteonecrosis (ON)

		ON confirmed by bone scan		Total
		(−)	(+)	
ON confirmed by MRI	(−)	31 (6.8)	8 (1.7)	39
	(+)	81 (17.7)	338 (80.7)	419
Total		112	346	458
ON confirmed by X-ray	(−)	72 (15.8)	58 (12.7)	130
	(+)	36 (11.0)	290 (63.6)	326
Total		108	348	456

κ = 0.325 between bone scan and MRI (*p* < 0.001); κ = 467 between bone scan and X-ray (*p* < 0.001)

We explored possible associations between WBBS uptake grade/defects and the ARCO classification, as determined by MRI (Table 6). The uptake grade was associated with the ARCO classification (*r* = 0.491, *p* < 0.001), but uptake defects were not (*r* = − 0.032, *p* = 0.496).

**Table 6:** Associations between uptake grade of, or defect evident on, whole-body bone scan (WBBS) and Association Research Circulation Osseous (ARCO) classifications on magnetic resonance imaging (MRI)

ARCO	Uptake grade of WBBS			Total	*p*-value	WBBS Photon defect		Total	*p*-value
	0	1	2			(−)	(+)		
0	32	5	2	39	**0.491,** **< 0.001**	5	34	39	−0.032, 0.496
1	30	11	17	58		14	44	58	
2	54	66	69	189		80	109	189	
3	9	33	101	143		39	104	143	
4	0	9	20	29		10	19	29	
Total	125	124	209	458		148	310	458	

*WBBS* whole-body bone scan, *ARCO* Association Research Circulation Osseous

## Discussion

We found that 10.2% of ON patients had multifocal ON associated with younger age, comorbidities, and the use of corticosteroids and immunosuppressive agents. Agreement between WBBS and MRI for all patients was fair, but the agreement between WBBS and X-rays was moderate. The ARCO score was significantly associated with the WBBS uptake grade.

Trauma and nontraumatic factors (excessive alcohol intake, corticosteroid use, and HIV infection) cause ON [2, 3]. Corticosteroid and alcohol use are responsible for more than 90% of all cases of ON [3]. Other causes include hypercoagulable conditions and radiation therapy [23]. We classified the causes of ON into four categories: idiopathic, trauma, comorbidities requiring the use of corticosteroids or immunosuppressive agents, and excessive alcohol intake. The principal causes were excessive alcohol intake (30.3%) and comorbidities requiring corticosteroid and/or immunosuppressive treatment (34.6%), similar to what was found in previous studies. Comorbidities included several diseases of the immune system, including SLE, dermatomyositis, and ulcerative colitis; malignancy; kidney transplantation to treat end-stage renal disease; and hematological conditions. We also included benign conditions requiring corticosteroids (aphthous ulcer, recurrent uveitis, facial palsy, and several allergic diseases) as comorbidities.

Multifocal ON is uncommon,being observed in only 3–11% of ON patients [5]. It was associated in several case studies and case reports with high-dose corticosteroid therapy, high alcohol intake, several immune system diseases (including SLE), organ transplantation, and malignancy [6, 8, 9, 12, 24]. Earlier studies were (prospective or retrospective) observational studies performed during treatment and follow-up in patients with sickle-cell disease, acute lymphocytic leukemia, and non-Hodgkin disease [25–27]. The incidence of multifocal ON was 44–82%. In this study, we did not evaluate the development of ON in patients at risk; rather, we found on review that 10.2% of 254 ON patients had multifocal ON as evidenced by WBBS and MR or X-ray images. Their mean age (42.8 ± 14.3 years) was less than that of patients with oligofocal ON (50.9 ± 15.4 years; *p* = 0.011). Furthermore, the most common causes of multifocal ON differed from those of oligofocal ON. Most former patients (69.2%) had comorbidities including systemic immune diseases, had undergone kidney transplantation, or had malignancies. Three patients consumed alcohol excessively, one patient had experienced severe trauma, and the causes of ON in four patients were unknown. We found that younger age, comorbidity, and use of corticosteroids and immunosuppressive agents were associated with multifocal ON. Several studies found that corticosteroid treatment, various rheumatic diseases, and hematological disease were so associated [5, 8, 11]. We derived ORs for the relevant factors: current corticosteroid use alone was highly significant in this context (OR = 4.512), but neither the cumulative nor maximal dose of corticosteroids differed between those with multifocal and oligofocal ON.

Conventional MRI is the most sensitive and specific imaging modality for early diagnosis and evaluation of ON progression [15]. However, the cost is high, and it is not yet covered by national health insurance in Korea. MRI can be used to evaluate one or two symptomatic lesions, but not all symptom-free areas can be examined, even if multifocal ON is suspected. A recent study assessed the utility of coronal, short-tau inversion recovery, whole-body MRI (STIR-WBMI) for evaluating ON in 15 patients with myositis [28]; STIR-WBMRI detected early multifocal ON. However, further studies with larger populations are needed. A recent study used whole-body MRI to evaluate 42 patients with Hodgkin lymphoma treated by chemotherapy. Of the 48 osteonecrotic lesions observed, 48% were detected in the knees, and multifocal ON was detected in six of seven patients (86%) [29]. Furthermore, the metadiaphysis was involved more frequently than the epiphysis (40% vs. 33%). As a result, whole-body MRI could be a very helpful tool for early detection of multifocal osteonecrotic lesions, but it is also expensive and is not yet covered by national health insurance in Korea. WBBS may be useful for diagnosing ON [16, 30]. WBBS is relatively inexpensive, and is easier to perform than MRI. However, several recent studies found that MRI was more sensitive [15, 31]. Another study found that WBBS was less sensitive than MRI for diagnosing symptomatic ON [1]. Although all 163 patients with histologically confirmed lesions were identified by MRI, only 56% were confirmed by WBBS. In our study, the extent of agreement between MRI and WBBS data was fair (κ = 0.325). However, 346 of 419 MRI-identified lesions were confirmed by WBBS (80.7%). Furthermore, the ARCO classification was significantly associated with femoral bone uptake grade on WBBS. In addition, WBBS identified 24 of 26 patients with multifocal ON (92.3%). Therefore, WBBS may be an additional tool for diagnosing ON and assessing its progression, especially in patients with suspected multifocal ON.

Our study has several limitations. First, this was a retrospective cross-sectional work. Second, we lacked bone biopsy data and instead used MRI or X-ray data to locate affected sites. Third, in patients with multifocal ON, not all ON sites were evaluated using MRI or X-ray. Fourth, selection bias may have been in play; we reviewed medical records. Corticosteroids are a major cause of ON. Therefore, patients treated with corticosteroids may undergo more WBBS than those not treated with corticosteroids, which may have resulted in selection bias in this study. Fourth, the ON sites were not evaluated according to the lesion location, such as the metadiaphyseal or epiphyseal regions, as described previously [29]. However, most lesions of the patients with ON were located in epiphyseal regions, and only seven patients had metadiaphyseal ON without epiphyseal ON. Twenty-three patients with ON had metadiaphyseal and epiphyseal ON. This was probably due to MRI being expensive in Korea, so that only the symptomatic sites were evaluated. However, this is the first study to calculate ORs for the risks associated with corticosteroid therapy and comorbidities in terms of multifocal ON, and to correlate quantitative bone scan data with the MRI ARCO classification of femoral head ON, which is representative of epiphyseal ON, in patients with ON. Our findings suggest that WBBS could play a useful role in the evaluation of multifocal ON.

## Conclusions

Multifocal ON is not uncommon. Age, comorbidity, and use of corticosteroids and immunosuppressive agents were significantly associated with multifocal ON. The ARCO classification correlated significantly with the bone uptake grade but not with photon defects. WBBS may be an additional tool for evaluating ON patients with risk factors for multiple ON, such as younger age, corticosteroid use, and comorbidities.

## Abbreviations

ARCO: Association Research Circulation Osseous
MRI: Magnetic resonance imaging
ON: Osteonecrosis
SD: Standard deviations
SLE: Systemic lupus erythematosus
STIR-WBMI: Short-tau inversion recovery, whole-body MRI
Tc-99 m HDP: Technetium-99 m hydroxymethylene diphosphonate
WBBS: Whole-body bone scan

## References

[1] Mont MA, Ulrich SD, Seyler TM, Smith JM, Marker DR, McGrath MS (2008). Bone scanning of limited value for diagnosis of symptomatic oligofocal and multifocal osteonecrosis. J Rheumatol.

[2] Assouline-Dayan Y, Chang C, Greenspan A, Shoenfeld Y, Gershwin ME (2002). Pathogenesis and natural history of osteonecrosis. Semin Arthritis Rheum.

[3] Ruderman M, McCarty DJ (1964). ARTHRITIS ROUNDS. (4). Aseptic necrosis in systemic lupus erythematosus. Report of a CASE involving six joints. Arthritis Rheum.

[4] Mankin HJ (1992). Nontraumatic necrosis of bone (osteonecrosis). N Engl J Med.

[5] LaPorte DM, Mont MA, Mohan V, Jones LC, Hungerford DS (1998). Multifocal osteonecrosis. J Rheumatol.

[6] Glueck CJ, Freiberg RA, Boppana S, Wang P (2008). Thrombophilia, hypofibrinolysis, the eNOS T-786C polymorphism, and multifocal osteonecrosis. J Bone Joint Surg Am.

[7] Sun W, Shi Z, Gao F, Wang B, Li Z (2016). The pathogenesis of multifocal osteonecrosis. Sci Rep.

[8] Jeong HJ, Kim D, Cho SK, Kim Y, Bae SC, Sung YK. Clinical characteristics of multifocal osteonecrosis in Korean patients with rheumatic disease. Int J Rheum Dis. 2018;21:1301–8. 10.1111/1756-185X.13065 28328178

[9] Flouzat-Lachaniete CH, Roussignol X, Poignard A, Mukasa MM, Manicom O, Hernigou P (2009). Multifocal joint osteonecrosis in sickle cell disease. Open Orthop J.

[10] Gomez-Puerta JA, Peris P, Reverter JC, Espinosa G, Martinez-Ferrer A, Monegal A (2013). High prevalence of prothrombotic abnormalities in multifocal osteonecrosis: description of a series and review of the literature. Medicine (Baltimore).

[11] Fajardo-Hermosillo LD, Lopez-Lopez L, Nadal A, Vila LM. Multifocal osteonecrosis in systemic lupus erythematosus: case report and review of the literature. BMJ Case Rep. 2013;2013:bcr2013008980. 10.1136/bcr-2013-008980 PMC3645777 23595183

[12] Mullan RH, Ryan PF (2002). Multiple site osteonecrosis in HIV infection. Rheumatology (Oxford).

[13] Mundo J, Peris P, Monegal A, Navasa M, Cervera R, Guaniabens N (2006). Multifocal avascular necrosis after liver transplantation: an unusual presentation of the antiphospholipid syndrome. Lupus.

[14] Grigoriu V, Stefaniu A, Fica N (1985). Evolution of a bronchial foreign body (a tooth chip). Rev Chir Oncol Radiol O R L Oftalmol Stomatol Otorinolaringol.

[15] Hauzeur JP, Pasteels JL, Schoutens A, Hinsenkamp M, Appelboom T, Chochrad I (1989). The diagnostic value of magnetic resonance imaging in non-traumatic osteonecrosis of the femoral head. J Bone Joint Surg Am.

[16] Alavi A, McCloskey JR, Steinberg ME. Early detction of avascular necrosis of the femoral head by 99m technetium diphosphonate bone scan: a preliminary report. Clin Orthop Relat Res. 1977;(127):137–41. 912968

[17] Conklin JJ, Alderson PO, Zizic TM, Hungerford DS, Densereaux JY, Gober A (1983). Comparison of bone scan and radiograph sensitivity in the detection of steroid-induced ischemic necrosis of bone. Radiology.

[18] Gregg PJ, Walder DN (1980). Scintigraphy versus radiography in the early diagnosis of experimental bone necrosis, with special reference to caisson disease of bone. J Bone Joint Surg Br.

[19] Minoves M, Riera E, Costansa JM, Bassa P, Setoain J, Domenech FM (1998). Multiple aseptic bone necrosis detected by Tc-99m MDP bone scintigraphy in a patient with systemic lupus erythematosus on corticosteroid therapy. Clin Nucl Med.

[20] Choi HR, Steinberg ME, Y Cheng E. Osteonecrosis of the femoral head: diagnosis and classification systems. Curr Rev Musculoskelet Med 2015; 8:210–220. 10.1007/s12178-015-9278-7 PMC4596207 26088795

[21] Osseous AARC. Committee on terminology and classification. ARCO News. 1992:41–6.

[22] Landis JR, Koch GG (1977). An application of hierarchical kappa-type statistics in the assessment of majority agreement among multiple observers. Biometrics.

[23] Jones JP Jr. Intravascular coagulation and osteonecrosis. Clin Orthop Relat Res. 1992:41–53. 1532547

[24] Symptomatic multifocal osteonecrosis. A multicenter study. Collaborative osteonecrosis group. Clin Orthop Relat Res. 1999;(369):312–26. 10611887

[25] Amin NL, Feltbower R, Kinsey S, Vora A, James B (2017). Osteonecrosis in patients with acute lymphoblastic leukaemia: a national questionnaire study. BMJ Paediatr Open.

[26] Rossleigh MA, Smith J, Straus DJ, Engel IA (1986). Osteonecrosis in patients with malignant lymphoma. A review of 31 cases. Cancer.

[27] Daltro G, Franco BA, Faleiro TB, Rosario DAV, Daltro PB, Fortuna V. Osteonecrosis in sickle cell disease patients from Bahia, Brazil: a cross-sectional study. Int Orthop. 2018;19:158. 10.1007/s00264-018-3905-z 29582115

[28] Zhen-Guo H, Min-Xing Y, Xiao-Liang C, Ran Y, He C, Bao-Xiang G (2017). Value of whole-body magnetic resonance imaging for screening multifocal osteonecrosis in patients with polymyositis/dermatomyositis. Br J Radiol.

[29] Albano D, Patti C, La Grutta L, Grassedonio E, Mulè A, Brancatelli G (2017). Osteonecrosis detected by whole body magnetic resonance in patients with Hodgkin lymphoma treated by BEACOPP. Eur Radiol.

[30] D'Ambrosia RD, Shoji H, Riggins RS, Stadalnik RC, DeNardo GL. Scintigraphy in the diagnosis of osteonecrosis. Clin Orthop Relat Res. 1978;(130):139–43. 639385

[31] Mitchell MD, Kundel HL, Steinberg ME, Kressel HY, Alavi A, Axel L (1986). Avascular necrosis of the hip: comparison of MR, CT, and scintigraphy. AJR Am J Roentgenol.

